# Correlations between Mortality-to-Incidence Ratios and Health Care Disparities in Testicular Cancer

**DOI:** 10.3390/ijerph17010130

**Published:** 2019-12-23

**Authors:** Wen-Jung Chen, Cheng-Yu Huang, Yu-Hui Huang, Shao-Chuan Wang, Tzuo-Yi Hsieh, Sung-Lang Chen, Wen-Wei Sung, Tsung-Hsien Lee

**Affiliations:** 1 Institute of Medicine, Chung Shan Medical University, Taichung 40201, Taiwan; mimic1024@gmail.com (W.-J.C.); yhhuang59@hotmail.com (Y.-H.H.); rosenbeck.wang@gmail.com (S.-C.W.); joe.hsieh46@gmail.com (T.-Y.H.); cshy650@csh.org.tw (S.-L.C.); 2 School of Medicine, Chung Shan Medical University, Taichung 40201, Taiwan; 3 Department of Urology, Chung Shan Medical University Hospital, Taichung 40201, Taiwan; 4 Department of Urology, National Taiwan University Hospital, Taipei 10002, Taiwan; 5 Department of Physical Medicine & Rehabilitation, Chung Shan Medical University Hospital, Taichung 40201, Taiwan; 6 Department of Obstetrics and Gynecology, Chung Shan Medical University, Taichung 40201, Taiwan; 7 Division of Infertility Clinic, Lee Women’s Hospital, Taichung 40652, Taiwan

**Keywords:** testicular cancer, mortality, incidence, mortality-to-incidence ratio

## Abstract

The mortality-to-incidence ratio (MIR) is associated with the clinical outcomes of different types of cancer as well as the ranking of health care systems. However, the association between MIRs for testicular cancer and health care disparities, including differences in expenditures and health system rankings, has not yet been reported. We used the Spearman’s rank correlation coefficient (CC) to analyze the correlation between testicular cancer MIRs and both total expenditures on health/gross domestic product (e/GDP) and the World Health Organization’s (WHO) health system rankings. After screening the data for quality and missing information, 57 countries were chosen for analysis. Generally, developed countries and regions had relatively high rates of incidence/mortality, but with a favorable MIR. Among the continents, Europe had the highest incidence rates, whereas the highest MIRs were in Africa. Globally, favorable testicular cancer MIRs were observed in countries with both a high e/GDP and a good WHO ranking (R^2^ = 0.325, *p* < 0.001 and CC = −0.568, *p* < 0.001; R^2^ = 0.367, *p* < 0.001 and CC = 0.655, *p* < 0.001, respectively). In conclusion, the MIR for testicular cancer varies in countries and regions based on both their total health expenditure and their health care system ranking.

## 1. Introduction

Testicular cancer is a relatively rare tumor type that comprises a mere 1% of all male cancers worldwide [1]. Testicular cancer not only has a distinctive age distribution, but is also a commonly diagnosed malignancy among men 15–40 years old [2]. Previous studies have predicted a steady rise in the cancer burden in the coming decades [3,4]. Despite an increase in its incidence, testicular cancer has the highest survival rate among male genital cancers: 95% of all patients will be cured, and they can expect long-term survival [5,6]. While cryptorchidism, family history, and ethnicity have been established as risk factors, the etiology of testicular cancer largely remains unclear [7].

In terms of geographic variation, the highest incidences of testicular cancer have been found in Western Europe (7.8%), Australia (6.5%), and North America (5.1%), where the Caucasian population predominates [8,9]. Nevertheless, the Africa region has the highest mortality-to-incidence ratio (MIR) [8]. In a large population-based analysis, non-Caucasians had poorer outcomes from testicular cancer (HR, 1.60; 95% CI, 1.22–2.10) [10] than did Caucasians. In addition to the disparity for ethnicity, previous studies in Africa have revealed that regions with high poverty, limited resources, and suboptimal health care systems face major challenges to disease management, which consequently lead to poorer prognoses [11]. Current MIRs can be calculated relatively easily from recent data sources. These data may be able to increase our understanding of those factors that lead to mortality rates that depart from incidence-based expectations [12].

Regarding socioeconomic status, a previously published review demonstrated that, for patients with a higher socioeconomic position, while the incidence of testicular cancer is higher, the survival rate is better than it is for patients with a lower socioeconomic status [13]. The World Health Organization’s (WHO) ranking of health systems has indicated an association between overall efficiency with resource inputs and the development ranking of a country, with industrialized countries dominant among the better performers [14]. However, some studies have provided no significant gradient for relative survival by deprivation category (1.3%; 95% CI, −0.3%–3.1%) [15]. Hence, the current inconsistencies imply the importance of deeply discerning the disparity in regions with different WHO rankings and socioeconomic statuses. In this study, we sought to analyze how regions with different WHO rankings and health care expenditures correlated with incidence and mortality rates for testicular cancer.

## 2. Methods

Cancer incidence and mortality data were obtained from the GLOBOCAN 2012 database, which is maintained by the International Agency for Research on Cancer (https://www.iarc.fr/). The WHO rankings were obtained from the World Health System’s report from the WHO, which was scored based on an index of factors including health, responsiveness, and fair financial contributions. Health expenditure and life expectancy data were obtained from the World Health Statistics of the WHO (https://www.who.int/). The percentage of total expenditures on health to gross domestic product (e/GDP) was calculated to indicate the total health expenditure. All data acquisition methods have been described previously [16,17].

The GLOBOCAN 2012 database contains data on 184 countries. We excluded countries with either missing or poor data quality. In total, 22 countries had no WHO ranking data. Up to 105 countries had low data availability, per the quality report of the GLOBOCAN 2012 database (either a ranking of E–G for incidence or a ranking of 4–6 for mortality). Ultimately, 57 countries were included in the analyses. The MIR has been defined previously as the ratio of the crude mortality rate to the incidence rate [16,18,19,20].

The statistical analysis methods that were used have been described previously [16,19,20,21]. We evaluated the association between the MIRs and the variables with the Spearman’s rank correlation coefficient (CC) using SPSS statistical software version 15.0 (SPSS, Inc., Chicago, IL, USA). *p* Values < 0.05 were considered statistically significant. Scatter plots were produced using Microsoft Excel 2016.

## 3. Results

### 3.1. Incidence and Mortality in Testicular Cancer Are Higher in More-Developed Regions than in Less-Developed Regions

To assess the global view of testicular cancer, we carried out an analysis of incidence and mortality based on region (Table 1). Overall, the world age-standardized rates (ASRs) for incidence and mortality were 1.5 and 0.3, respectively. Among more-developed regions, the ASR for incidence was higher than it was in less-developed regions (incidence: 5.2 vs 0.7, respectively). Nevertheless, there was no significant difference revealed in the ASR for mortality (mortality: 0.3 vs 0.3, respectively). Concerning the analysis that was based on WHO regions and continents, the ASR for incidence in the WHO’s European region and the continent of Europe exceeded those of other areas. Nevertheless, there was a gradual convergence of the ASR for mortality among all WHO regions and continents (ASR mortality: 0.1–0.5). These results indicate that testicular cancer in regions with high development and in Europe had higher incidence rates of testicular cancer, but there was a smaller difference for mortality.

### 3.2. The Mortality-to-Incidence Ratios for Prostate and Testicular Cancer Are High in Less-Developed Regions

Because the MIR has been proven useful for identifying disparities among cancers, regional differences in cancer MIRs were investigated. The world MIR for testicular cancer was 0.19. Contrary to the findings in the crude rate, both the less-developed regions and the regions with lower human development had higher MIRs (0.38 and 0.07, respectively) than other regions. Furthermore, higher MIRs were noted in the WHO’s East Mediterranean region, Africa region, and South-East Asia region (0.56, 0.50, and 0.50, respectively). Regarding continents, Africa had the highest MIR (0.67). Therefore, for the MIR of testicular cancer, less-developed regions, the East Mediterranean region, and the Africa region possessed higher MIRs.

### 3.3. A Country’s World Health Organization Ranking and Total Expenditure on Health/Gdp Have a Significant Association with Its Mortality-to-Incidence Ratio for Testicular Cancer

To further assess the epidemiologic differences among countries, we analyzed the countries of this study based on their position in the WHO’s rankings (Table 2). The information on the WHO ranking, the e/GDP, and life expectancy are also shown in Table 2. Among the included countries, France had the highest WHO ranking, and the United States of America had the highest e/GDP (17.0%). As for the ASR of incidence and mortality, the highest incidence was in Norway (12.2), and the highest mortality was in Chile (1.0). However, as for the MIR of individual countries, the Philippines, Fiji, and the South African Republic possessed the highest ratio (0.50).

**Figure 1:**
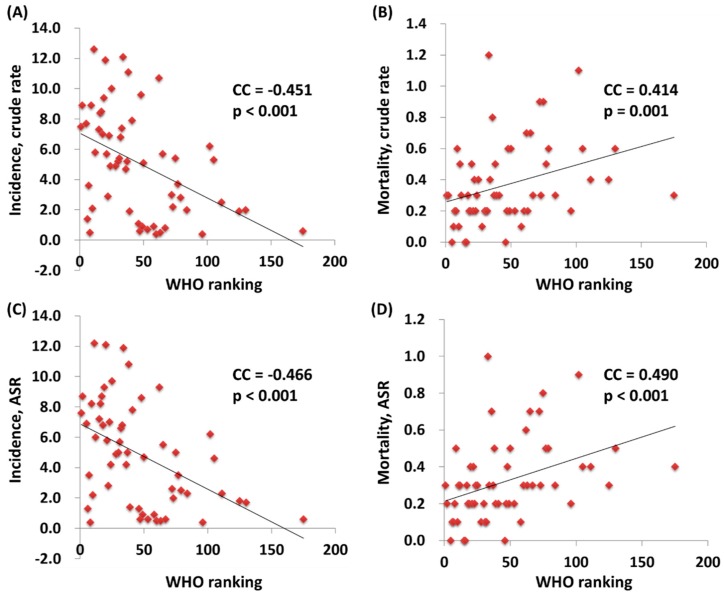
The association between the World Health Organization’s rankings and crude rates of (**A**) incidence and (**B**) mortality, and the ASR (age-standardized rate) of (**C**) incidence and (**D**) mortality.

**Figure 2:**
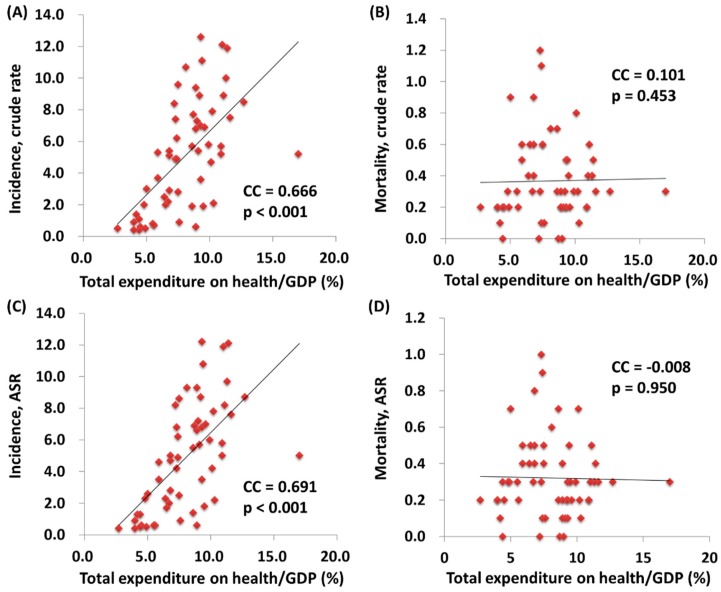
The association between the total expenditures on health/GDP and the crude rates of (**A**) incidence and (**B**) mortality, and the ASR (age-standardized rate) of (**C**) incidence and (**D**) mortality.

**Figure 3:**
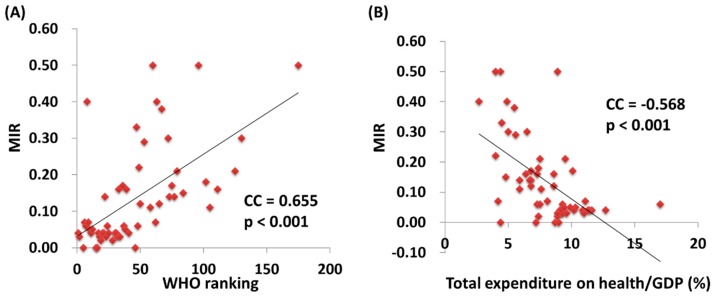
The World Health Organization’s (**A**) rankings and (**B**) total expenditures on health/GDP are significantly associated with the MIR in testicular cancer.

We further correlated the WHO ranking and the e/GDP with the ASR and MIR of testicular cancer by country. The WHO ranking reversely correlated to the e/GDP (R^2^ = 0.109, *p* = 0.12 and CC = −0.463, *p* < 0.001). Countries with a better WHO ranking had a higher crude rate and a higher ASR for incidence (R^2^ = 0.200, *p* < 0.001 and CC = −0.451, *p* < 0.001; R^2^ = 0.214, *p* < 0.001 and CC = −0.466, *p* < 0.001, respectively; Figure 1). Likewise, a higher crude rate and a higher ASR for incidence were noticed in countries with a higher e/GDP (R^2^ = 0.364, *p* < 0.001 and CC = 0.666, *p* < 0.001; R^2^ = 0.383, *p* < 0.001 and CC = 0.691, *p* < 0.001, respectively; Figure 2). Countries with a better WHO ranking had a lower crude rate and a lower ASR for mortality (R^2^ = 0.109, *p* = 0.012 and CC = 0.414, *p* = 0.001; R^2^ = 0.150, *p* = 0.003 and CC = 0.490, *p* < 0.001, respectively; Figure 1). Nevertheless, no statistically significant difference for either the crude rate or the ASR of mortality was shown among the countries with different e/GDPs (R^2^ < 0.001, *p* = 0.897 and CC = 0.101, *p* = 0.454; R^2^ < 0.001, *p* = 0.885 and CC = −0.008, *p* = 0.950, respectively; Figure 2). Regarding the MIR, a better WHO ranking and a higher e/GDP were associated with a favorable MIR (number of countries = 57; R^2^ = 0.367, *p* < 0.001 and CC = 0.655, *p* < 0.001; R^2^ = 0.325, *p* < 0.001 and CC = −0.568, *p* < 0.001, respectively; Figure 3), illustrating that a country’s WHO ranking and e/GDP are significantly associated with its MIR for testicular cancer. Furthermore, to confirm the influence of countries with a relatively small incidence or mortality case number, we analyzed their correlation after including a criteria of incidence or mortality case number larger than 10. The results in countries with incidence numbers larger than 10 continued to show that a better WHO ranking and a higher e/GDP were associated with a favorable MIR (number of countries = 52, R^2^ = 0.470, *p* < 0.001 and CC = 0.728, *p* < 0.001; R^2^ = 0.269, *p* < 0.001 and CC = −0.565, *p* < 0.001, respectively). As for those with mortality number larger than 10, a better WHO ranking and a higher e/GDP were associated with a favorable MIR (number of countries=37, R^2^ = 0.471, *p* < 0.001 and CC = 0.754, *p* < 0.001; R^2^ = 0.402, *p* < 0.001 and CC = –0.719, *p* < 0.001, respectively), as expected.

## 4. Discussion

Testicular cancer is a relatively indolent disease. A high five-year survival rate of 74% with distant metastasis is significantly higher than the 6% for non-small cell lung cancer. This can be reflected by differences in MIRs. Globally, the MIR of lung cancer is 0.87 [21], while it is 0.19 for testicular cancer. Despite that, our study found a significant correlation between the MIR for testicular cancer and a country’s WHO ranking, echoing the findings of previous papers on colon cancer and lung cancer [18,21]. Therefore, it is clear that better health care programs can provide better detection and treatment for testicular cancer.

Unlike colon cancer, there is neither a standard nor a routine screening for testicular cancer. Furthermore, incidences of this disease are growing, and it is the most common malignancy in males aged 15–45 years [22]. Hence, a major part of the endeavor to increase detection must rely on public education to raise personal awareness. A study that was conducted in Northern Ireland found that only 17% of men within the at-risk age range had heard of a testicular self-examination, suggesting the urgent need for a more aggressive promotion of awareness by the health care system [23]. When considering that the United Kingdom has one of the lowest MIRs at 0.03, one can only imagine the scarcity of personal awareness promotion programs and education that are provided in countries with high MIRs.

Although most cases of testicular cancer are detected by a physical examination and a medical history check, a considerable proportion are detected incidentally. A retrospective study that was conducted in 2004 found that 11 of 150 patients who had undergone orchiectomy for testicular cancer had their cancer detected by a scrotal sonography examination that was intended for an infertility evaluation [24]. This technique enables the early diagnosis of small nonpalpable tumors. Such examinations may not be accessible in countries with poorer WHO rankings, which thus diminishes the possibility for earlier detection. A study that was conducted in Tanzania in 2014 found a stunning 39.3% of patients with stage IV diseases at presentation [11]. The treatment for testicular cancer consists of radical orchidectomy, cisplatin-based chemotherapy, radiotherapy, and retroperitoneal lymph node dissection. However, cisplatin-based chemotherapy is not routinely used in many African countries [11]. The higher costs of such regimens and a lack of adequate support from health care systems may contribute to such a phenomenon.

There are some limitations to our study. For example, we excluded countries with either poor data quality or little data from this study to avoid the possible production of misleading MIRs. However, this may lead to incompleteness in the data collection and a reduction in the generalizability of the results. Regardless of the consideration of data quality, as all countries are included, favorable testicular cancer MIRs were still observed in countries with both a high e/GDP and a good WHO ranking (N = 142, R^2^ = 0.138, *p* < 0.001 and CC = −0.391, *p* < 0.001; N = 145, R^2^ = 0.465, *p* < 0.001 and CC = 0.678, *p* < 0.001, respectively). Moreover, important risk factors for testicular cancer, such as undescending testis and infertility, were not documented. Another drawback is that this ecological study only analyzed data for one year, which may not have been enough to accurately reflect real trends for this disease. The generalizability of its findings at the individual level from the country level is the major limitation of this study. Also, the actual role of MIR is still conflicted, since the MIR would never replace the role of survival data from cohort survey [25]. Furthermore, since both the WHO ranking and e/GDP are indicators of infrastructure needed for cancer care, no causal relationship of these factors to the MIR was concluded.

Despite these limitations, our study was the first to identify a correlation between health care ranking and MIR for testicular cancer, suggesting that both the detection and the management of such an indolent disease can be examined and evaluated by MIRs. Although this is a novel indicator, we can also use it to assess the quality and accessibility of a health care system while urging worldwide advancement in medical practices.

## 5. Conclusions

A favorable MIR for testicular cancer was related to a high percentage of a country’s GDP going toward health care and a good ranking of their health care system. This makes it a potentially useful score for the global evaluation of health care systems and outcomes for testicular cancer.

## Figures and Tables

**Table 1:** Summary of the case numbers, rates, and MIRs of the incidence and mortality of testicular cancer per region.

Region	Number		Crude Rate ^1^		Age-Standardized Rate ^1^		Mortality-to-Incidence Ratio ^2^
	Incidence	Mortality	Incidence	Mortality	Incidence	Mortality	
World	55,266	10,351	1.6	0.3	1.5	0.3	0.19
Development							
More-developed regions	32,740	2209	5.4	0.4	5.2	0.3	0.07
Less-developed regions	22,526	8142	0.8	0.3	0.7	0.3	0.38
WHO region categories							
WHO Africa region	1024	593	0.2	0.1	0.3	0.2	0.50
WHO Americas region	16,162	1988	3.4	0.4	3.2	0.4	0.12
WHO East Mediterranean region	2843	1438	0.9	0.5	0.9	0.5	0.56
WHO Europe region	23,560	2302	5.4	0.5	5	0.4	0.09
WHO South-East Asia region	5854	2766	0.6	0.3	0.6	0.3	0.50
WHO Western Pacific region	5819	1264	0.6	0.1	0.6	0.1	0.17
Continent							
Africa	1529	864	0.3	0.2	0.4	0.3	0.67
Latin America and Caribbean	7197	1504	2.4	0.5	2.2	0.5	0.21
Northern America	8965	484	5.2	0.3	5	0.2	0.06
Asia	15,053	5849	0.7	0.3	0.7	0.3	0.43
Europe	21,548	1612	6	0.5	5.6	0.4	0.08
Oceania	974	38	5.2	0.2	5	0.2	0.04

MIRs: mortality-to-incidence ratios; ^1^ Rates were defined as the rates per 100,000. ^2^ The percentage in the ratio of the crude rate of mortalities and the crude rate of incidences.

**Table 2:** Summary of selected countries’ World Health Organization rankings, total expenditures on health/GDP, life expectancies, testicular cancer incidences, mortality, and MIRs.

Country	Ranking	Total Expenditure on Health/GDP (%)	Life Expectancy	Number		Crude Rate ^1^		Age-Standardized Rate ^1^		Mortality-to-Incidence Ratio ^2^
				Incidence	Mortality	Incidence	Mortality	Incidence	Mortality	
France	1	11.6	82	2332	98	7.5	0.3	7.6	0.3	0.04
Italy	2	9.2	83	2664	82	8.9	0.3	8.7	0.2	0.03
Malta	5	8.7	81	16	0	7.7	0.0	6.9	0.0	0.00
Singapore	6	4.2	83	37	3	1.4	0.1	1.3	0.1	0.07
Spain	7	9.3	83	823	42	3.6	0.2	3.5	0.1	0.06
Oman	8	2.7	76	8	3	0.5	0.2	0.4	0.2	0.40
Austria	9	11.1	81	368	25	8.9	0.6	8.2	0.5	0.07
Japan	10	10.3	84	1274	86	2.1	0.1	2.2	0.1	0.05
Norway	11	9.3	82	313	12	12.6	0.5	12.2	0.3	0.04
Portugal	12	9.9	81	302	18	5.8	0.3	6.0	0.3	0.05
Iceland	15	9.0	82	12	0	7.3	0.0	7.2	0.0	0.00
Luxembourg	16	7.2	82	22	0	8.4	0.0	8.2	0.0	0.00
Netherlands	17	12.7	81	709	26	8.5	0.3	8.7	0.3	0.04
United Kingdom	18	9.3	81	2163	64	7.0	0.2	6.8	0.2	0.03
Ireland	19	8.9	81	216	5	9.4	0.2	9.3	0.2	0.02
Switzerland	20	11.4	83	453	19	11.9	0.5	12.1	0.4	0.04
Belgium	21	10.9	80	300	13	5.7	0.2	5.8	0.2	0.04
Colombia	22	6.8	78	676	89	2.9	0.4	2.8	0.4	0.14
Sweden	23	9.6	82	329	9	6.9	0.2	7.0	0.2	0.03
Cyprus	24	7.3	82	28	2	4.9	0.3	4.2	0.3	0.06
Germany	25	11.3	81	4031	146	10.0	0.4	9.7	0.3	0.04
Israel	28	7.4	82	185	5	4.9	0.1	4.9	0.1	0.02
Canada	30	10.9	82	890	36	5.2	0.2	5.0	0.2	0.04
Finland	31	9.1	81	144	4	5.4	0.2	5.7	0.1	0.04
Australia	32	8.9	83	780	20	6.8	0.2	6.6	0.1	0.03
Chile	33	7.3	80	640	101	7.4	1.2	6.8	1.0	0.16
Denmark	34	11.0	80	336	11	12.1	0.4	11.9	0.3	0.03
Costa Rica	36	10.1	79	114	19	4.7	0.8	4.2	0.7	0.17
United States of America	37	17.0	79	8073	448	5.2	0.3	5.0	0.3	0.06
Slovenia	38	9.4	80	111	5	11.1	0.5	10.8	0.5	0.05
Cuba	39	8.6	78	106	17	1.9	0.3	1.4	0.2	0.16
New Zealand	41	10.2	82	173	7	7.9	0.3	7.8	0.2	0.04
Bahrain	46	4.4	77	9	0	1.1	0.0	1.3	0.0	0.00
Thailand	47	4.5	75	208	64	0.6	0.2	0.6	0.2	0.33
Czech Republic	48	7.5	78	496	30	9.6	0.6	8.6	0.4	0.06
Malaysia	49	4.0	74	135	34	0.9	0.2	0.9	0.2	0.22
Poland	50	6.8	77	939	116	5.1	0.6	4.7	0.5	0.12
Jamaica	53	5.6	74	9	3	0.7	0.2	0.6	0.2	0.29
Korea, Republic of	58	7.6	82	222	17	0.9	0.1	0.9	0.1	0.11
Philippines	60	4.4	69	204	94	0.4	0.2	0.5	0.3	0.50
Slovakia	62	8.1	76	284	18	10.7	0.7	9.3	0.6	0.07
Egypt	63	4.9	71	204	97	0.5	0.2	0.5	0.3	0.40
Uruguay	65	8.6	77	93	12	5.7	0.7	5.5	0.7	0.12
Trinidad and Tobago	67	5.5	71	5	2	0.8	0.3	0.6	0.3	0.38
Belarus	72	5.0	72	131	41	3.0	0.9	2.6	0.7	0.30
Lithuania	73	6.7	74	34	5	2.2	0.3	2.0	0.3	0.14
Argentina	75	6.8	76	1090	184	5.4	0.9	5.0	0.8	0.17
Estonia	77	5.9	77	23	3	3.7	0.5	3.5	0.5	0.14
Ukraine	79	7.5	71	570	133	2.8	0.6	2.5	0.5	0.21
Mauritius	84	4.8	74	13	2	2.0	0.3	2.3	0.3	0.15
Fiji	96	4.0	70	2	1	0.4	0.2	0.4	0.2	0.50
Bulgaria	102	7.4	75	220	41	6.2	1.1	6.2	0.9	0.18
Latvia	105	5.9	74	55	6	5.3	0.6	4.6	0.4	0.11
Ecuador	111	6.4	76	187	31	2.5	0.4	2.3	0.4	0.16
Brazil	125	9.5	75	1873	364	1.9	0.4	1.8	0.3	0.21
Russian Federation	130	6.5	69	1330	399	2.0	0.6	1.7	0.5	0.30
South African Republic	175	8.9	60	151	68	0.6	0.3	0.6	0.4	0.50

^1^ Rates were defined as the rates per 100,000. ^2^ The percentage in the ratio of the crude rate of mortalities and the crude rate of incidences.

## Abbreviations

ASR	age-standardized rate
CC	correlation coefficient
e/GDP	total expenditures on health/gross domestic product
MIR	mortality-to-incidence ratio
WHO	World Health Organization
